# Sildenafil Ameliorates Long Term Peripheral Neuropathy in Type II Diabetic Mice

**DOI:** 10.1371/journal.pone.0118134

**Published:** 2015-02-17

**Authors:** Lei Wang, Michael Chopp, Alexandra Szalad, LongFei Jia, XueRong Lu, Mei Lu, Li Zhang, Yi Zhang, RuiLan Zhang, Zheng Gang Zhang

**Affiliations:** 1 Department of Neurology, Henry Ford Hospital, 2799 W. Grand Boulevard, Detroit, Michigan, 48202, United States of America; 2 Department of Physics, Oakland University, Rochester, Michigan, 48309, United States of America; Hosptial Infantil Universitario Niño Jesús, CIBEROBN, SPAIN

## Abstract

Diabetic peripheral neuropathy is a common complication of long-standing diabetes mellitus. To mimic clinical trials in which patients with diabetes enrolled have advanced peripheral neuropathy, we investigated the effect of sildenafil, a specific inhibitor of phosphodiesterase type 5 enzyme, on long term peripheral neuropathy in middle aged male mice with type II diabetes. Treatment of diabetic mice (BKS.Cg-*m*+/+*Lepr^db^/J*, db/db) at age 36 weeks with sildenafil significantly increased functional blood vessels and regional blood flow in the sciatic nerve, concurrently with augmentation of intra-epidermal nerve fiber density in the skin and myelinated axons in the sciatic nerve. Functional analysis showed that the sildenafil treatment considerably improved motor and sensory conduction velocities in the sciatic nerve and peripheral thermal stimulus sensitivity compared with the saline treatment. In vitro studies showed that mouse dermal endothelial cells (MDE) cultured under high glucose levels exhibited significant down regulation of angiopoietin 1 (Ang1) expression and reduction of capillary-like tube formation, which were completely reversed by sildenafil. In addition, incubation of dorsal root ganglia (DRG) neurons with conditioned medium harvested from MDE under high glucose levels suppressed neurite outgrowth, where as conditional medium harvested from MDE treated with sildenafil under high glucose levels did not inhibit neurite outgrowth of DRG neurons. Moreover, blockage of the Ang1 receptor, Tie2, with a neutralized antibody against Tie2 abolished the beneficial effect of sildenafil on tube formation and neurite outgrowth. Collectively, our data indicate that sildenafil has a therapeutic effect on long term peripheral neuropathy of middle aged diabetic mice and that improvement of neurovascular dysfunction by sildenafil likely contributes to the amelioration of nerve function. The Ang1/Tie2 signaling pathway may play an important role in these restorative processes.

## Introduction

Diabetic peripheral neuropathy is a common complication of diabetes. There is a compelling need to develop therapeutic approaches for diabetic peripheral neuropathy. Although numerous reagents have been validated in experimental diabetic peripheral neuropathy, clinical trials show that the majority of them do not achieve clinical benefits for treatment of diabetic peripheral neuropathy [1,2]. Generally, young diabetic animals with an early stage of peripheral neuropathy are employed in experiments to investigate efficacy of compounds that are of therapeutic interest, whereas patients with diabetes enrolled in clinical trials often have advanced peripheral neuropathy; these differences in age and stage of diabetic neuropathy may contribute to the failure of clinical trials.

Cyclic guanosine monophosphate (cGMP) is generated by cytoplasmic soluble guanylatecyclases, and the phosphodiesterase type 5 (PDE5) enzyme is highly specific for hydrolysis of cGMP [3,4]. Among its functions, cGMP regulates vascular function, axon guidance and synaptic plasticity [5]. Experimental data show that treatment of diabetic peripheral neuropathy with a potent PDE5 inhibitor, sildenafil, improves blood supply to the sciatic nerve [6,7]. Patients with diabetic peripheral neuropathy who are treated for erectile dysfunction with sildenafil have reduced symptoms of neuropathy [8]. We previously demonstrated that hyperglycemia upregulated PDE5 expression, and suppression of PDE5 by sildenafil increased cGMP levels and significantly ameliorated peripheral neuropathy in diabetic mice [9]. However, it is not known whether the therapeutic effect of sildenafil can be achieved in diabetic mice with long term peripheral neuropathy, because diabetic mice used in our previous study were relatively young, i.e.16 weeks old. To mimic the clinical situation, in the present study, we treated 36 week old diabetic mice with sildenafil.

Vascular dysfunction is closely coupled with neuronal damage and precedes impairment of sciatic nerve damage in diabetic peripheral neuropathy [10–12]. The angiopoietins (Ang) and their receptor Tie-2 regulate vascular stabilization and maturation [13,14]. The Ang/Tie2 signaling pathway promotes neurite outgrowth in cultured dorsal root ganglion (DRG) neurons [15]. Sildenafil-induced angiogenesis is mediated by Ang1 [16]. However, whether the Ang1/Tie2 pathway is involved in diabetic peripheral neuropathy has not been fully investigated.

Here, we report that sildenafil ameliorates peripheral neuropathy in diabetic mice at the age of 36 weeks, and that the Ang1/Tie2 signaling pathway likely underlies the beneficial effects of sildenafil on neurovascular function in diabetic mice.

## Materials and Methods

### Ethics Statement

All experimental procedures were carried out in accordance with NIH Guidelines for the Care and Use of Laboratory Animals and were approved by the Institutional Animal Care and Use Committee of Henry Ford Hospital (IACUC #1185). Male BKS. Cg-*m*+/+*Lepr* ^*db*^ */J* (db/db) mice (Jackson Laboratories) aged 36 weeks were used. Age-matched male heterozygote mice (db/m), a non-penetrant genotype (Jackson Laboratories), were used as the control animals.

### Sildenafil treatment

db/db mice at the age of 36 weeks were treated with sildenafil at a dose of 10 mg/kg (orally administered, o.p. Viagra, Pfizer Inc.), every day for 8 weeks (n = 15/group). db/db mice (n = 15/group) at the same age treated with the same volume of saline were used as a control group. Age-matched db/m mice treated with sildenafil (10 mg/kg o.p. treated daily, n = 15/group) or saline (n = 15/group) were used as additional control groups. All mice were sacrificed 8 weeks after treatment. Doses of sildenafil were selected based on published studies [9].

Levels of blood glucose, triglyceride, and A1C were measured using an instant check meter (Roche Diagnostics), CardioChekPA Analyzer and Triglyceride Test Strips (Polymer 285 Technology system), and A1C Now+ MULTI-TEST A1C SYSTEM, respectively, according to the manufacturer’s instructions. Blood glucose levels, body weight and functional tests were measured before the treatment as a baseline and then every 2 weeks until sacrifice. Triglyceride and A1C levels were measured prior to the treatment and at the end of the experiment (8 weeks after the initial treatment). Electrophysiological measurements and functional tests were performed before the treatment and then every 4 weeks until sacrifice. All procedures and analyses were performed by investigators who were blinded to the treatment administered.

### Neurophysiological Measurements

Sciatic nerve conduction velocity was assessed with orthodromic recording techniques, as previously described [9,17]. Briefly, mice were anesthetized with ketamine/xylazine (i.p., 100/10 mg/kg). The stimulating electrodes were plated at the knee and sciatic notch. Trigger single square wave current pulses were delivered using an isolated pulse stimulator (Model 2100, A-M Systems). The simultaneous electromyographies were recorded by two sterilized electrodes placed in the dorsum of the foot with a Grass Amplifier (Model P5, Grass Instruments). During the measurements, animal rectal temperature was maintained at 37 ± 1.0°C using a feedback controlled water bath. Motor nerve conduction velocity (MCV) and sensory nerve conduction velocity (SCV) were calculated according to a published study [17].

### Measurement of thermal sensitivity

To examine the sensitivity to noxious heat, plantar and tail flick tests were measured using a thermal stimulation meter (IITC model 336 TG combination tail-flick and paw algesia meter; IITC Life Science) according to published methods [18]. Briefly, mice were placed within a plexiglass chamber on a transparent glass surface and allowed to acclimate for at least 20 min. For plantar test, the meter was activated after placing the stimulator directly beneath the plantar surface of the hind paw. The paw-withdrawal latency in response to the radiant heat (15% intensity, cut-off time 30 sec) was recorded. For tail-flick test, the meter was set at 40% heating intensity with a cut-off at 10 sec. For both tests, at least five readings per animal were taken at 15 min intervals, and the average was calculated [19].

### Measurement of regional blood flow by laser Doppler flowmetry

Regional blood flow in the sciatic nerve was measured at the end of the experiments (8 weeks after the treatment) using laser Doppler flowmetry (LDF PeriFlux PF4, Perimed AB) [19,20]. Briefly, under anesthesia (ketamine/xylazine, i.p., 100/10 mg/kg, JHP Pharmaceuticals LLC.; LLOYD Inc.), the mouse was mounted on a Kopf stereotaxic apparatus. The left sciatic nerve was exposed in the mid-thigh region and animal rectal temperature was maintained at 37 ± 1.0°C during the measurement period using a feedback controlled water bath. Using a micromanipulator, a LDF probe was placed at the surface of the sciatic nerve and relative flow values expressed as perfusion units were recorded every 5 minutes for a total of 5 records. Regional blood flow values from db/m mice were used as baseline values and data are presented as a percentage of baseline values.

### Measurement of microvascular perfusion

To examine microvascular perfusion in the sciatic nerve, fluorescein isothiocyanate (FITC)-dextran (2×10^6^ molecular weight, Sigma; 0.2 ml of 50 mg/ml) was administered intravenously to the mice 10 min before sacrifice [19,21]. Sciatic nerve tissue was rapidly removed and fixed in 2% of paraformaldehyde for 2 hours. Whole mount preparation of the nerve tissue was performed and FITC-dextran perfused vessels in the whole mount were imaged under a 10x objective using a laser-scanning confocal microscope (Zeiss LSM 510 NLO, Carl Zeiss, Inc.) [19,21]. Thereafter, the nerve tissue was embedded in OCT compound and cross cryosections (20 μm thickness) were cut.

### Staining myelin sheets

The sciatic nerves were fixed in the 2.5% glutaraldehyde and 0.5% sucrose (Sigma) on PBS buffer for 6–8 hours, and then immersed in 2% osmium tetroxide (Sigma) for 2 hours. The specimens were then dehydrated with numerous alcohol passages and embedded in paraffin [22]. Semi-thin transverse sections (2-μm thick) were cut and stained with 1% toluidine blue and three semi-thin sections per mouse were analyzed.

### Immunohistochemistry

The sciatic nerves were fixed in 4% paraformaldehyde for immunohistochemistry and then embedded in paraffin according to published protocol [9]. Three cross sections (6-μm-thick) or three longitudinal sections (6-μm-thick) at 60 μm apart per animal were used [9].

Footpads were fixed in Zamboni’s fixative for 2 hours, washed in PBS and then kept in 30% sucrose/PBS overnight at 4°C. The samples were embedded in OCT compound and stored at-80°C. Three longitudinal 20 μm-thick footpad sections from each mouse were prepared.

The following primary antibodies were used: polyclonal rabbit anti-Ang1 (1:2000, Abcam), monoclonal mouse anti-CD31 antibody (1:500, BD Biosciences), polyclonal rabbit anti-S100 (1:400, Abcam) and polyclonal rabbit anti-protein gene product 9.5 (PGP9.5, 1:1000, MILLIPORE). Rabbit or goat IgG was used as a negative control. Sections were counterstained with 4′, 6-Diamidino-2-phenylindole (DAPI) (1:5000, Thermo Scientific).

### Image acquisition and quantification

Image analysis was performed using a computer imaging analysis system (MicroComputer Imaging Device, MCID, Imaging Research Inc.) [23].

To examine microvascular perfusion in the sciatic nerve, three sections at 60 μm intervals from each mouse were used for further image analysis. The cross sections were digitized under a 20x microscope objective (Carl Zeiss, Inc.) via a MCID system. The total number of FITC-dextran perfused vessels was counted and divided by the total tissue-area to determine vascular density [19].

For analysis of CD31 immunoreactive vascular morphology and perimeter, three cross sections spaced at 60 μm intervals from each mouse were used. Three fields of the view per section were randomly imaged under a 20x objective. CD31 immunoreactive vascular perimeters were measured using MCID system [19].

For morphometric analysis of sciatic nerves, three sections spaced as 60 μm interval for each staining were used for analysis from each mouse and three fields of the view per section were randomly imaged under a 100x oil immersion objective (BX40; Olympus Optical Co. Ltd). Myelinated fiber diameter, axon diameter, and myelin sheath thickness were measured. The g ratio (the quotient axon diameter/fiber diameter) was calculated to measure the degree of myelination. At least 200 myelinated fibers were measured per animal [9,24].

Intraepidermal nerve fiber profiles were digitized under a 40x objective (Carl Zeiss, Inc.) via the MCID system. The number of nerve fibers crossing the dermal-epidermal junction were counted and the density of nerves are expressed as fibers/mm length of section [25].

All analysis was conducted with the examiner who was blinded to the identity of the samples being studied.

### Cell culture

A regular glucose medium (RG) was defined as a medium containing 5 mM glucose, while a high glucose medium (HG) was referred to as a medium containing 30 mM glucose, which was chosen to match glucose levels prevalent in uncontrolled diabetic patients [26]. These glucose concentrations have been used for the in vitro hyperglycemia experiments by others [27,28].

To examine the effect of sildenafil on endothelial and Schwann cells, Mouse Dermal Microvascular Endothelial Cells (MDE, Cell Biologics Inc.) and Mouse Schwann Cells (MSC, ScienCell Research Laboratories), respectively, were cultured according to the manufactures’ instructions.

To assess the effect of sildenafil on in vitro angiogenesis, a capillary-like tube assay was used [29–31]. Briefly, MDE were cultured under regular or high glucose condition in the presence or absence of sildenafil (300 nM) for 48 hours. MDE (2x10^4^ cells) were cultured on 96-well plate coated with Matrigel (BD Biosciences) for 5 hours. Total length of tubes was measured in 3 random fields from each well using MCID system [32]. Experiments were independently repeated 6 times (n = 6/group).

### Conditioned media

To collect conditioned medium from MDE, 2.5 × 10^6^ cells were plated onto a 100-mm-diameter dish in 10 ml of defined medium. The cells were cultured under the regular or high glucose condition in the presence or absence of sildenafil (300 nM) for 48 hours and the supernatant (conditioned medium) was collected. The conditioned media were concentrated 10 times using 10 kD centrifugal filters (Amicom Ultra-15; Nihon Millipore), and frozen at −80°C until use.

Primary culture of DRG neurons and evaluation of neurite outgrowth: DRG neurons were harvested from 18–20 week old male db/m mice. Cultures were prepared according to a previously described procedure with some modifications [33]. Briefly, DRGs were removed and stripped of meninges, and dissociated by a combination of Ca^2+^- and Mg^2+^- free Hanks balance salt solution (HBSS) containing 0.125% trypsin and 0.1% collagenase—A digestion for 30 min, then mechanically triturated for ~20 times. Isolated DRG neurons were cultured in Neurobasal-A medium (Invitrogen), 5 mM glucose, 2% B-27, 1% Pen/Strep/Neo, and 1%GlutamAX, and 10 nM uridine and 10 nM 5-flurodeoxyuridine.

To evaluate the effects of conditional medium harvested from endothelial cells treated with sildenafil on neurite outgrowth, DRG neurons were plated at 2,000 cells/well in a 24 well-plate, containing coverslips coated by laminin in DRG culture medium with one-tenth the endothelial cells conditioned medium. After 3 days in culture, DRG neurons were immunofluorescently stained with antibodies against neurofilament heavy-chain (NFH, 1:1000, Covance). NFH immunoreactive neurites of 20 individual DRG neurons per coverslip were imaged under a 20x objective. The neurite length of each neuron was measured using MCID system [34]. The average length of neurite outgrowth was calculated. Data are presented as ratio to control. Experiments were independently repeated 6 times (n = 6/group).

### Schwann cell migration

To examine the effect of endothelial cells treated with sildenafil on migration of Schwann cells, a modified Boyden’s chamber assay was employed, as described previously [35]. Briefly, the polycarbonate filter (8-μm pore size) (Neuro Probe, Inc.) was coated by 50 μg/ml fibronectin (Chemicon) and 0.1% gelatin (Sigma) and placed between upper and lower chambers. Schwann cells (5x10^4^ cells per well) were placed in the upper chamber in the presence or absence of a neutralizing antibody against Tie2, and the lower chamber was filled with endothelial cell conditioned medium. The chamber was incubated for 5 hours at 37°C and 5% CO_2_. Migrating cells caught in the membrane were then stained using hematoxylin and eosin (Anatech Ltd). The numbers of cells that migrated through the filter were counted in 5 fields of view under a 40X objective. Experiments were independently repeated 6 times (n = 6/group).

### Western blot analysis

Western blot was performed according to published methods [36]. Briefly, equal amounts of proteins were loaded on 10% SDS-polyacrylamide gel. After electrophoresis, the proteins were transferred to nitrocellulose membranes, and the blots were subsequently probed with the following antibodies: polyclonal rabbit anti-Ang1 (1:1000, Abcam). For detection, horseradish peroxidase-conjugated secondary antibodies were used (1:2000) followed by enhanced chemiluminescence development (Pierce). Normalization of results was ensured by running parallel Western blot with β-actin antibody. The optical density was quantified using an image processing and analysis program (Scion Image). Experiments were independently repeated 6 times (n = 6/group).

### Statistical analysis

For functional tests, data were evaluated for normality. Ranked data or nonparametric approach will be considered if the data are not normally distributed. The repeated measure analysis of variance (ANOVA) was considered with dependent factor of time and independent factor of groups. The analysis started testing for group by time interaction, followed by the testing the main effect of group and subgroup analyses. Two-sample t-test or analysis of variance (ANOVA) was used to study the group difference on LDF, immunostaining, biochemistry, Western blot, and tube formation analysis, respectively. The data are presented as mean ± SE. A value of *p*<0.05 was taken as significant.

## Results

### Sildenafil improves neurological outcome in diabetic mice with long-term diabetic neuropathy

**Fig 1:**
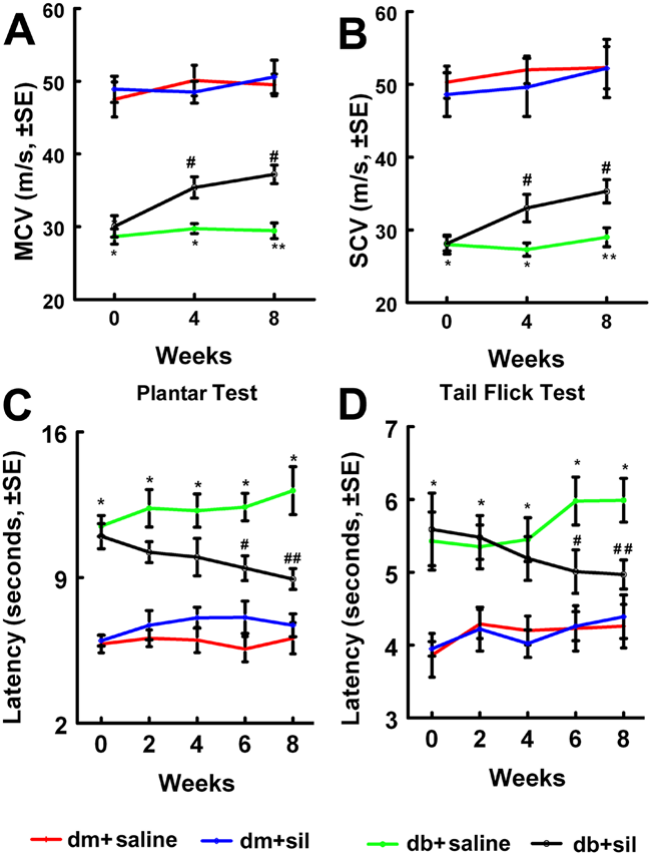
Sildenafil improves neurological function in db/db mice. Treatment of male db/db mice with sildenafil improves neurological function measured by MCV (A), SCV (B), Plantar test (C) and Tail flick test (D). *p<0.05, **p<0.01 versus the non-diabetic mouse (dm) and #p<0.05, ##p<0.01 versus the diabetic mouse (db) treated with saline, respectively. n = 10/group.

Treatment of male db/db mice at age 16 weeks with sildenafil is effective in ameliorating peripheral neuropathy [9]. To examine the therapeutic effect of sildenafil on db/db mice with long-term peripheral neuropathy, sildenafil was administered at a dose of 10 mg/kg to male db/db mice at middle age of 36 weeks, and treated daily for 8 consecutive weeks, and the mice were sacrificed at age 44 weeks. We found that sildenafil treatment significantly improved diabetes—reduced motor and sensory conducting velocity (MCV and SCV) in the sciatic nerve measured by electrophysiological tests (Fig. 1A, B). The thermal latency with plantar test and tail flick test revealed that sildenafil markedly improved sensory function starting at 6 weeks after treatment compared with saline-treated db/db mice (Fig. 1C, D). Treatment of the db/db mouse with sildenafil did not significantly alter blood glucose levels, A1C, triglyceride and animal body weight (Table 1, 2 and 3). These data indicate that sildenafil improves neurological function even in middle aged mice with long-term diabetic peripheral neuropathy.

**Table 1:** Effect of sildenafil on body weight.

Body Weight (g)					
Groups	0 w	2 w	4 w	6 w	8 w
**dm-saline**	**36.0±0.9**	**35.0±1.1**	**35.0±0.9**	**34.3±1.1**	**33.4±1.09**
**dm-sil**	**36.2±1.2**	**36.3±0.9**	**36.5±0.9**	**36.4±0.8**	**35.1±0.8**
**db-saline**	49.0±3.2*	42.5±3.2*	42.1±3.5*	42.0±3.5*	45.4±3.2*
**db-sil**	51.8±2.4*	49.8±2.7*	46.7±2.3*	45.6±2.2*	47.0±1.9*

Values are mean±SE.

*p<0.01 versus dm-saline group. n = 10/group. W = week, 0 w represents before the treatment, while other numbers indicate after the treatment. dm = non-diabetic mouse; db = diabetic mouse; sil = sildenafil.

**Table 2:** Effect of sildenafil on blood glucose.

Blood glucose(g/dl)					
Groups	0 w	2 w	4 w	6 w	8 w
**dm-saline**	**151±9.7**	**132±4.1**	**125±7.8**	**127±8.5**	**153±7.8**
**dm-sil**	**140±4.6**	**137±4.1**	**132±5.4**	**148±5.2**	**163±10.6**
**db-saline**	556±14.0*	548±17.1*	551±12.1*	525±20.1*	515±47.6*
**db-sil**	537±22.7*	579±9.9*	509±36.7*	479±31.6*	555±26.1*

Values are mean±SE.

*p<0.01 versus dm-saline group. n = 10/group. W = week, 0 w represents before the treatment, while other numbers indicate after the treatment. dm = non-diabetic mouse; db = diabetic mouse; sil = sildenafil.

**Table 3:** Effect of sildenafil on AIC and Triglyceride.

	AIC		Triglyceride	
Groups	0 w	8 w	0 w	8 w
**dm-saline**	**4.3±0.06**	**4.2±0.05**	**58.2±2.8**	**51.0±1.0**
**dm-sil**	**4.4±0.06**	**4.3±0.07**	**50.2±0.2**	**52.89±1.5**
**db-saline**	11.1±0.55*	10.6±0.44*	78.3±4.7*	84.5±8.0*
**db-sil**	10.4±0.31*	10.1±0.61*	72.8±4.7*	87.3±6.0*

Values are mean±SE.

*p<0.01 versus dm-saline group. n = 10/group. W = week, 0 w represents before the treatment, while other numbers indicate after the treatment. dm = non-diabetic mouse; db = diabetic mouse; sil = sildenafil.

### Sildenafil improves neurovascular function

**Fig 2:**
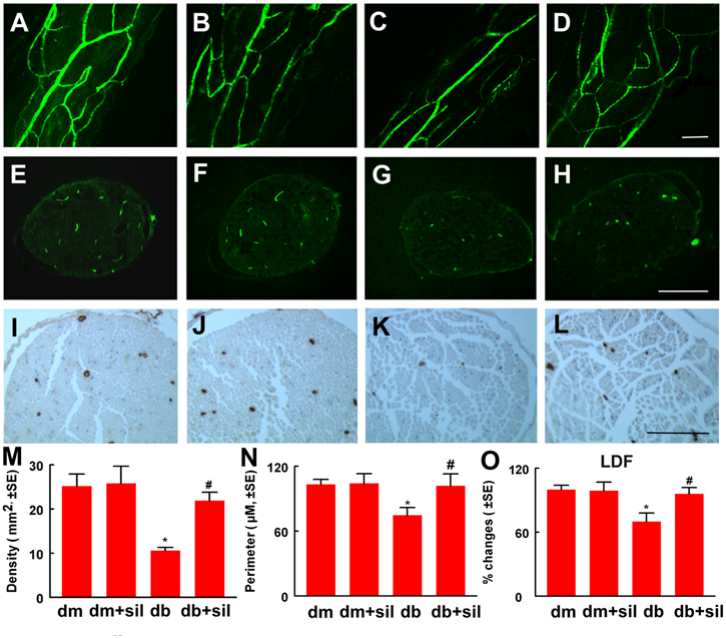
Sildenafil improves neurovascular function in the sciatic nerve. Panels A to L show FITC-dextran perfused vessels from whole mounted (A to D) and cross sections (E to H) of the sciatic nerve, and CD31 immunoreactive blood vessels at the cross section (I and L) of the sciatic nerve from a representative non-diabetic mouse (dm) treated with saline (A,E and I), dm mouse treated with sildenafil (dm+sil, B,F and J), diabetic mouse (db) treated with saline (C,G and K) and db mouse treated with sildenafil (db+sil, D,H and L). Panels M to O show quantitative data of density of FITC-dextran perfused vessels in cross section (M, n = 5/group), CD31 immunoreactive vascular perimeters (N, n = 10/group), and percentage changes of blood flow in the sciatic nerve with a reference of dm mice at 100% (O, n = 10/group). *p<0.05 and #p<0.05 versus the saline treated non-diabetic mouse (dm) and diabetic mouse (db), respectively. Bar = 100 μm.

Moreover, we found that treatment of middle aged diabetic mice with sildenafil significantly increased local blood flow in the sciatic nerve, as measured by LDF (Fig. 2O). In parallel with blood flow results, analysis of FITC-perfused blood vessels in 3D images acquired from whole mount of the sciatic nerve revealed that diabetes induced substantial reduction of FITC-perfused blood vessels compared to that in non-diabetic mice, whereas treatment of diabetic mice with sildenafil significantly increased the number of FITC-dextran perfused vessels (Fig. 2A to D). In addition, treatment of diabetic mice with sildenafil increased microvascular density and vascular perimeters (Fig. 2E to N). Collectively, these data indicate that sildenafil improves blood vascular perfusion in the sciatic nerves of diabetic mice.

**Fig 3:**
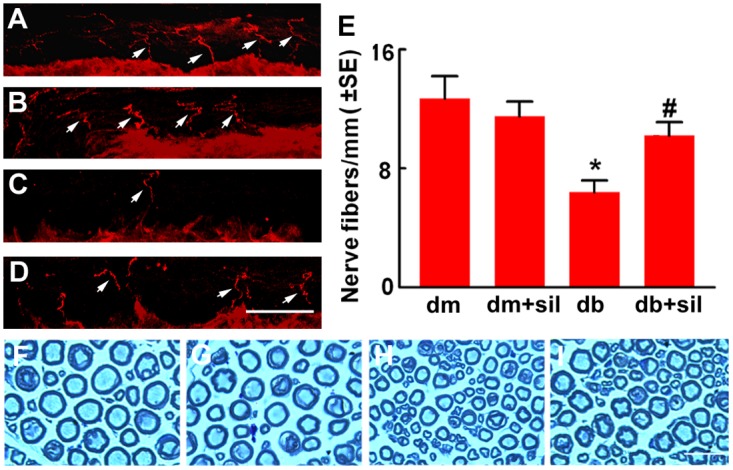
Sildenafil increases axonal remodeling in db/db mice. Panels A to D show PGP 9.5 immunoreactive *epidermal nerve fibers* (red, arrows) in the plantar skin from a representative non-diabetic mouse treated with saline (dm, A), non-diabetic mouse treated with sildenafil (dm+sil, B), diabetic mouse treated with saline (db, C) and diabetic mouse treated with sildenafil (db+sil, D). Panel E shows quantitative data. Panels F to I show semi-thin toluidine blue-stained cross sections of sciatic nerves from a representative non-diabetic mouse (F), non-diabetic mouse treated with sildenafil (G), diabetic mouse treated with saline (H), and diabetic mouse treated with sildenafil (I). Values are mean±SE. *p<0.05, **p<0.01 versus non-diabetic mouse (dm) treated with saline and #p<0.05, ##p<0.01 versus diabetic mouse (db) treated with saline. n = 10/group. Bar in D = 10 μm and Bar in I = 20 μm.

To examine whether the sildenafil-improved blood perfusion affects the sciatic nerves, intraepidernal nerve fiber density on plantar skin tissue was measured. Diabetic mice exhibited robust reduction of nerve fibers, which is consistent with findings from skin biopsy sample from diabetic patients showing that reduction of blood vessels is closely associated with axonal degeneration [10]. Sildenafil substantially increased nerve fiber density in diabetic mice compared to saline treatment (Fig. 3A to E). Analysis of myelinated axons of the sciatic nerve showed that sildenafil considerably augmented myelin thickness and the g ratio, but did not significantly alter axon diameters compared to saline treatment (table 4). These data indicate that sildenafil-improved vascular perfusion is associated with enhancement of axonal myelination and skin nerve fiber density.

**Table 4:** Effect of sildenafil on histomorphometric parameters of sciatic nerves.

Property	Dm		Db	
	+saline	+sildenafil	+saline	+sildenafil
**Fiber diameter(μm)**	**8.98±0.11**	**9.03±0.11**	7.96±0.13**	**8.34±0.07** ^**#**^
**Axon diameter(μm)**	**5.65±0.06**	**5.66±0.08**	5.25±0.1**	**5.14±0.04**
**Myelin thickness(μm)**	**1.67±0.05**	**1.68±0.04**	1.35±0.05**	**1.60±0.03** ^**##**^
**G ratio**	**0.63±0.01**	**0.62±0.01**	0.66±0.01*	**0.62±0.01** ^**##**^

Values are mean±SE.

*p<0.05,

**p<0.01 versus dm-saline group and

#p<0.05,

##p<0.01 versus db-saline group. n = 10/group. W = week, 0 w represents before the treatment, while other numbers indicate after the treatment. dm = non-diabetic mouse; db = diabetic mouse; sil = sildenafil.

### The Ang1/Tie2 signaling pathway mediates the beneficial effect of sildenafil on neurovascular function

**Fig 4:**
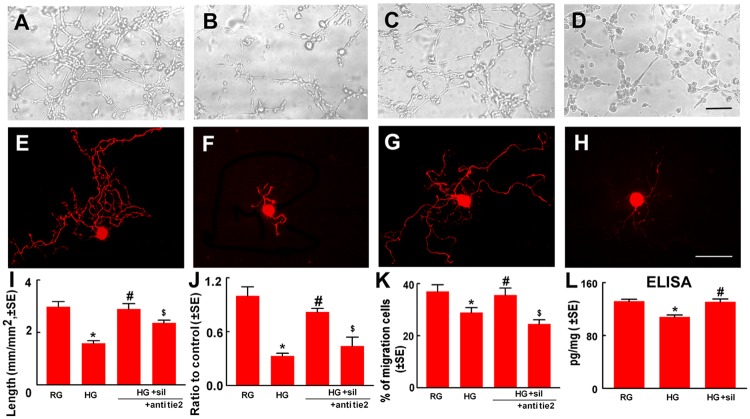
Sildenafil induces in vitro angiogenesis, DRG neurite outgrowth and Schwann cell migration. Representative microscopic images (A to D) and quantitative data (I) show capillary-like tube formation of endothelial cells under regular glucose (RG), high glucose (HG), high glucose with sildenafil (HG+sil, 300 nM), and high glucose with sildenafil in the presence of a neutralizing antibody against Tie2 (+Tie2, 5 μg/ml). Panels E to H show NFH immunoreactive DRG neuron cultured in conditional medium derived from endothelial cells treated with regular glucose (RG), high glucose (HG), high glucose with sildenafil (HG+sil, 300 nM), and high glucose with sildenafil in the presence of a neutralizing antibody against Tie2 (+Tie2, 5 μg/ml). Panels J and K show quantitative data of neurite outgrowth from DRG neurons and the percentage of Schwann cell migration under different conditions listed above. Panel L shows ELISA data of Ang1 levels in conditional medium harvested from endothelial cells cultured with regular glucose (RG), high glucose (HG), high glucose with sildenafil (+sil, 300 nM). *p<0.05 versus regular glucose (RG), and #p<0.05 versus high glucose (HG), respectively. n = 6/group. Bar in D = 50 μm and Bar in H = 20 μm.

To examine molecular mechanisms underlying the effect of sildenafil on neurovascular function, we performed in vitro experiments. Use of a capillary-like tube formation assay, an in vitro angiogenesis assay, we first examined the effect of sildenafil on endothelial cells. Compared to regular glucose conditions, high glucose conditions considerably reduced endothelial cells to form capillary tubes, whereas sildenafil reversed high glucose-reduced capillary tube formation (Fig. 4A to D and I). These data indicate that sildenafil can overcome the effect of high glucose induced endothelial cell dysfunction.

We then examined whether alteration of endothelial cell function by high glucose and sildenafil affects DRG neurons and Schwann cells. DRG neurons were cultured with conditional medium harvested from endothelial cells under regular glucose, high glucose, or high glucose with sildenafil, and neurite outgrowth were measured. Compared to the regular glucose conditional medium, the high glucose conditional medium substantially reduced neurite outgrowth, while the conditional medium from high glucose with sildenafil did not block neurite outgrowth (Fig. 4E to H and J). In addition, the high glucose conditional medium suppressed Schwann cell migration compared to the regular glucose conditional medium (Fig. 4K). Inhibition of Schwann cell migration was not detected under the high glucose with sildenafil conditional medium (Fig. 4K). These data suggest that soluble factors secreted by endothelial cells in conditional medium affect functions of DRG neurons and Schwann cells.

**Fig 5:**
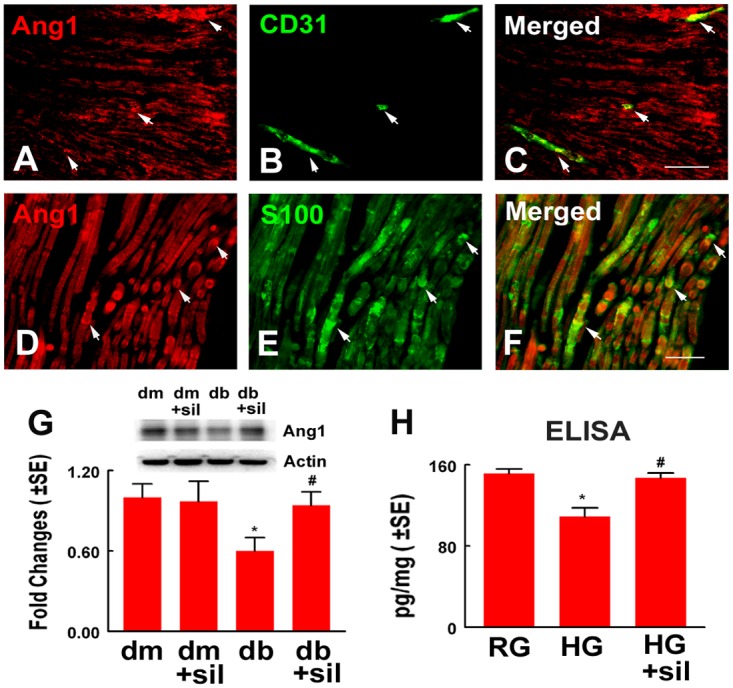
The Ang1/Tie2 signaling pathway mediates the effect of sildenafil on neurovascular function. Representative images of double immunofluorescent staining show that Ang1 immunoreactivity (A, C, D, F, red, arrows) was co-localized to CD31 positive vessels (B, C, green, arrows) and S100 positive Schwann cells (E, F, green, arrows). Western blot analysis (G) shows Ang1 levels in sciatic nerve tissue and β-actin was used as an internal control. ELISA data (H) show Ang1 levels in conditional medium harvested from Schwann cells cultured with regular glucose (RG), high glucose (HG), high glucose and sildenafil (+sil, 300 nM). *p<0.05 versus non-diabetic mouse (dm) treated with saline and regular glucose (RG). #p<0.05 versus diabetic mouse (db) treated with saline and high glucose (HG), respectively. n = 6/group. Bar = 50 μm.

Ang1 is an endothelial cell secreted soluble protein, and has been shown to enhance neurite growth of neurons [15]. Using an ELISA, Ang1 levels were measured in medium harvested from endothelial cells cultured under different conditions. The presence of Ang1 proteins was detected in the regular glucose medium, whereas Ang1 protein levels were significantly reduced in high glucose medium (Fig. 4L). However, endothelial cells treated with sildenafil under high glucose conditions secreted Ang1 proteins at the level comparable to the regular glucose medium (Fig. 4L). These data suggest that Ang1 plays a role in mediating the effect of sildenafil-treated endothelial cells on DRG neurons and Schwann cells. Accordingly, we blocked the Ang1 receptor, Tie2, with a neutralizing antibody against Tie2. In the presence of the neutralized antibody against Tie2, the effect of sildenafil on neurite outgrowth and Schwann cell migration under high glucose conditional medium harvested from endothelial cells was abolished (Fig. 4J, K). Moreover, Western blot analysis of sciatic nerve tissue revealed that diabetic mice had a significant reduction of Ang1 proteins compared to non-diabetic mice, while diabetic mice treated with sildenafil had Ang1 proteins comparable to non-diabetic mice (Fig. 5G). Double immunofluorescent staining showed that endothelial cells and Schwann cells in the sciatic nerve tissue were Ang1 positive (Fig. 5A to F). Interestingly, cultured Schwann cells also expressed Ang1 (Fig. 5H). Sildenafil attenuated high glucose reduced Ang1 level measured by ELISA (Fig. 5H). Collectively, our data indicate the Ang1/Tie2 signaling pathway activated by sildenafil mediates improvement of neurovascular function in diabetic peripheral neuropathy.

## Discussion

In the present study, we provide evidence that the sildenafil treatment improves neurological outcome even in middle age diabetic mice with long term peripheral neuropathy. Enhancement of peripheral neurovascular function by sildenafil likely contributes to amelioration of peripheral neuropathy, while the Ang1/Tie2 signaling pathway may mediate the therapeutic effect of sildenafil.

Diabetic neuropathy progresses from initial functional to late structural changes [2]. Diabetic db/db mice develop peripheral neuropathy characterized by slowed sciatic nerve conduction velocity at age of 14 weeks, while morphometric changes including myelinated and unmyelinated axons occur approximately at age of 20 weeks [37], which resemble patients with diabetic peripheral neuropathy [38]. Therapies targeting neurovascular function have been shown to restore nerve function in experimental diabetic peripheral neuropathy [17,39]. Treatment of diabetic peripheral neuropathy with sildenafil improves blood supply to the sciatic nerve and functional recovery [6,40]. Data from a case report study showed that patients with diabetes treated with sildenafil for erectile dysfunction exhibit amelioration of peripheral neuropathy [8]. We previously demonstrated that treatment of db/db mice at age of 16 weeks with sildenafil significantly improves neurological function [9]. The present study confirms and extends our previous findings by showing that the treatment of diabetic mice with sildenafil starting at age of 36 weeks substantially increased functional vascular density and regional blood flow in the sciatic nerve, and improved sciatic nerve conduction velocities and sensory function, although, significant improvement of sensory function was not observed until 6 weeks after the initial treatment. The present findings suggest that sildenafil could have potential clinical application for patients with long term diabetic peripheral neuropathy because patients with diabetes enrolled in clinical trials often have advanced peripheral neuropathy [2].

Vascular dysfunction leads to nerve damage [10,12]. Neurovascular dysfunction including axonal atrophy and segmental demyelination is a major cause of diabetic peripheral neuropathy [41]. Clinically, skin biopsy is widely used to evaluate intraepidermal *nerve* fibers for diagnosis of neuropathy [42]. The present study showed that db/db mice at age 44 weeks exhibited substantial reduction of intraepidermal nerve fiber density and decreases of axon diameter and myelin thickness in the sciatic nerve. Sildenafil-improved vascular function was closely associated with increases in intraepidermal nerve fiber density and sciatic nerve myelin thickness, but failed to show improvement in axon atrophy. Moreover, our in vitro data indicate that endothelial cells activated by sildenafil suppress the inhibitory effect induced by high glucose on DRG neurite outgrowth and Schwann cell migration. Collectively, our data support that enhancement of vascular function facilitates axonal regeneration and remyelination [10].

Compared to the treatment of diabetic mice with sildenafil starting at age of 16 weeks [9], sildenafil initiating at animal age of 36 weeks did not robustly augment axon diameter of sciatic nerves. Although we do not know exact causes to the failure to regenerate atrophied axons by sildenafil, we speculate that the success of axonal remodeling process may be dependent on the intensity of damage and therapy duration. Axonal damage induced by diabetes may be irreversible at age of 36 weeks. In addition, an eight week course of treatment may not be sufficient to document a significant increase in the axonal diameter of the sciatic nerve and middle aged diabetic mice with long term neuropathy may decrease in their ability to respond to sildenafil treatment. Preclinical and clinical studies have demonstrated that the late phases of diabetic neuropathy are poorly reversible, and *early intervention is* an important determinant of outcome in diabetic neuropathy [2].

Impairment of the Ang/Tie2 signaling pathway contributes to development of diabete-induced vascular dysfunction [43]. Ang1 not only regulates vascular function, but also directly promotes neurite outgrowth in DRG neurons [15]. Our in vivo and in vitro data suggest that the Ang/Tie2 signaling pathway mediates sildenafil-improved neurovascular function. We first demonstrated that hyperglycemia considerably reduced Ang1 levels in endothelial cells and Schwann cells, which is consistent with published studies showing that hyperglycemia decreases Ang1 levels in the animal model of diabetic myocardial infarction [44]. In addition, the present study showed that sildenafil upregulated Ang1 expression in endothelial and Schwann cells under hyperglycemia condition, while Ang1 secreted by sildenafil-activated endothelial cells enhanced neurite outgrowth of DRG neurons. More importantly, blockage of Ang/Tie2 signaling attenuated therapeutic effect of sildenafil on endothelial cells, Schwann cells and DRG neurons under hyperglycemia condition. These data support a view that the Ang/Tie2 signaling pathway mediates common cues that govern vascular and nervous systems.

In summary, our data demonstrate that treatment of middle age male diabetic mice with sildenafil is effective to ameliorate peripheral neuropathy by improvement of peripheral neurovascular function. The Ang1/Tie2 signaling pathway plays an important role in these processes. These findings provide new insights into mechanisms underlying the neurological dysfunction of long term diabetic peripheral neuropathy and may lead to the development of a sildenafil therapy for long term diabetic peripheral neuropathy.
